# Effects of JPEG Compression on Vision Transformer Image Classification for Encryption-then-Compression Images

**DOI:** 10.3390/s23073400

**Published:** 2023-03-23

**Authors:** Genki Hamano, Shoko Imaizumi, Hitoshi Kiya

**Affiliations:** 1 Graduate School of Science and Engineering, Chiba University, 1-33 Yayoicho, Chiba 263-8522, Japan; 2 Graduate School of Engineering, Chiba University, 1-33 Yayoicho, Chiba 263-8522, Japan; 3 Faculty of System Design, Tokyo Metropolitan University, 6-6 Asahigaoka, Tokyo 191-0065, Japan

**Keywords:** JPEG compression, vision transformer, encryption-then-compression system, encrypted domain, image classification

## Abstract

This paper evaluates the effects of JPEG compression on image classification using the Vision Transformer (ViT). In recent years, many studies have been carried out to classify images in the encrypted domain for privacy preservation. Previously, the authors proposed an image classification method that encrypts both a trained ViT model and test images. Here, an encryption-then-compression system was employed to encrypt the test images, and the ViT model was preliminarily trained by plain images. The classification accuracy in the previous method was exactly equal to that without any encryption for the trained ViT model and test images. However, even though the encrypted test images can be compressible, the practical effects of JPEG, which is a typical lossy compression method, have not been investigated so far. In this paper, we extend our previous method by compressing the encrypted test images with JPEG and verify the classification accuracy for the compressed encrypted-images. Through our experiments, we confirm that the amount of data in the encrypted images can be significantly reduced by JPEG compression, while the classification accuracy of the compressed encrypted-images is highly preserved. For example, when the quality factor is set to 85, this paper shows that the classification accuracy can be maintained at over 98% with a more than 90% reduction in the amount of image data. Additionally, the effectiveness of JPEG compression is demonstrated through comparison with linear quantization. To the best of our knowledge, this is the first study to classify JPEG-compressed encrypted images without sacrificing high accuracy. Through our study, we have come to the conclusion that we can classify compressed encrypted-images without degradation to accuracy.

## 1. Introduction

Significant advances in deep-learning technology have made it possible to automate and accelerate various tasks. In particular, image classification has been put to practical use in a variety of applications, such as face recognition and anomaly detection. In addition, cloud services have become popular among common organizations and individuals for the purpose of reducing costs, facilitating data sharing, and so on. For these reasons, image classification tasks are increasingly being accomplished through cloud servers. To utilize a model on a cloud server, a user should transmit test images to the server.

However, cloud servers are generally not reliable, and thus test images are under threat of being compromised outside of the servers. As a result, the copyright and privacy information in the test images might be disclosed to third parties. Additionally, the user generally needs to classify a large number of images; in other words, a large amount of image data should be transmitted to the server in succession. Therefore, it is desirable to minimize the data amount of the test images.

Many compression methods have been proposed to reduce the data amount of images. Compression methods can be classified into two categories: lossless and lossy methods. In general, lossy methods can more efficiently reduce the data amount compared with lossless methods. A typical lossy method is JPEG, which is one of the image compression standards. On the basis of human visual features, JPEG compression significantly reduces the information of high-frequency components and commonly applies 4:2:0 downsampling, i.e., horizontal and vertical downsampling of chrominance. Consequently, we can notably reduce the data amount while preserving high image quality. It is noted that JPEG compression can adjust the image quality and data amount by varying the quality factor.

In recent years, there has been a great amount of effort to develop secure image-classification systems with copyright and privacy protection for images. Federated learning is one technique that can be used in developing such systems [1,2,3]. Multiple clients individually train a single model by using their own data, while a central server integrates the parameters trained by each client. This technique can protect training images but not test images. On another front, secure computation is also drawing attention. This technique can directly adapt computational operations to encrypted data. A large number of methods have been proposed that automatically classify data encrypted with secure computation [4,5,6]. These methods can protect test data; however, the encrypted data can hardly be compressed. Even if the encrypted data is successfully compressed, it is difficult to decrypt the data.

Another approach for protecting copyright and privacy information in test images is to conceal the visual information. Image encryption is a typical technique for concealing visual information, and image-encryption methods have been actively studied to train encrypted images using deep neural networks [3,7,8,9,10,11,12,13,14,15,16,17]. The method in [3] combines federated learning with image encryption for test images. Encrypted image classification via a cloud server assumes that a user encrypts test images and transmits the encrypted images to a server. Thus, it is desirable to be able to compress the encrypted images in terms of the transmission efficiency; however, most such methods [3,9,10,11,12,13,14,15,16] do not consider image compression. Aprilpyone et al. employed the encryption-then-compression (EtC) system [18] as an image encryption algorithm so that the encrypted images (hereafter, EtC images) possess a high compression performance [8]. Some other methods protect visual information using machine learning instead of encryption and classify protected images [19,20]. The methods [8,9,10,11,12,13,14,15,19,20], however, degrade the classification accuracy due to the protection of visual information.

The method in [8] employs the Vision Transformer (ViT) [21] and ConvMixer [22], which are called isotropic networks, as image-classification models. They are known to provide a higher classification accuracy compared with convolutional neural networks, which are the conventional mainstream image-classification models. Kiya et al. focused on the properties of ViT to maintain the classification accuracy for encrypted images [16]. This method prepares a series of encryption keys (hereafter, key set) and uses it to encrypt not only test images but also a trained ViT model. The encrypted ViT model is eventually suitable for the encrypted images. This is the first study that perfectly preserves the classification accuracy for encrypted images. However, the image encryption process in this method employs a pixel-wise transformation, so the encrypted images can hardly be compressed.

As an extension of the method [16], we previously introduced an EtC system for the image encryption process [17]. The EtC system is based on a block-wise transformation, and thus the EtC images can maintain high compression performance. Further, this method does not cause any degradation to the classification accuracy for EtC images by using a model encryption algorithm that corresponds to the EtC system. Therefore, we not only successfully avoid any degradation to the classification accuracy but also compress the encrypted images. In [17], we surveyed the performance of lossless compression using JPEG-LS [23].

On the basis of our previous method [17], this paper, for the first time, investigates the effects of JPEG compression, which is a widely used lossy compression standard, on the classification accuracy. In our experiments, we confirm that a high classification accuracy can be preserved even for JPEG-compressed EtC images. Moreover, this paper verifies the effectiveness of JPEG compression in terms of classification and compression performance compared with linear quantization.

In this paper, we demonstrate that JPEG noise added to the high-frequency component barely degrades the accuracy of ViT classification. To the best of our knowledge, this is the only study that successfully compresses encrypted images using the JPEG lossy standard and classifies the compressed encrypted-images with very little degradation to accuracy. Through a series of studies, we reach the conclusion that compressed EtC images can be classified without degradation to accuracy.

## 2. Preparation

We give an overview of ViT [21] and summarize our previous method [17] in this section. We previously proposed an image classification method using ViT with novel advantages; copyrights for both a trained ViT model and test images can be protected simultaneously without any decrease in the accuracy of classification, and the test images are effectively compressed using lossless image compression standards. This paper verifies the effects of JPEG lossy compression on the classification accuracy of ViT on the basis of our previous method.

### 2.1. Vision Transformer

An attention mechanism dynamically identifies the location that should be focused on within input data. This mechanism has notably contributed to enhancing accuracy in deep learning. In the field of natural-language processing, there is a transformer in which an attention mechanism is implemented that enhances the performance of machine translation [24]. By using the transformer for image classification tasks, ViT has achieved higher accuracy than with conventional methods, such as convolutional neural networks.

**Figure 1:**
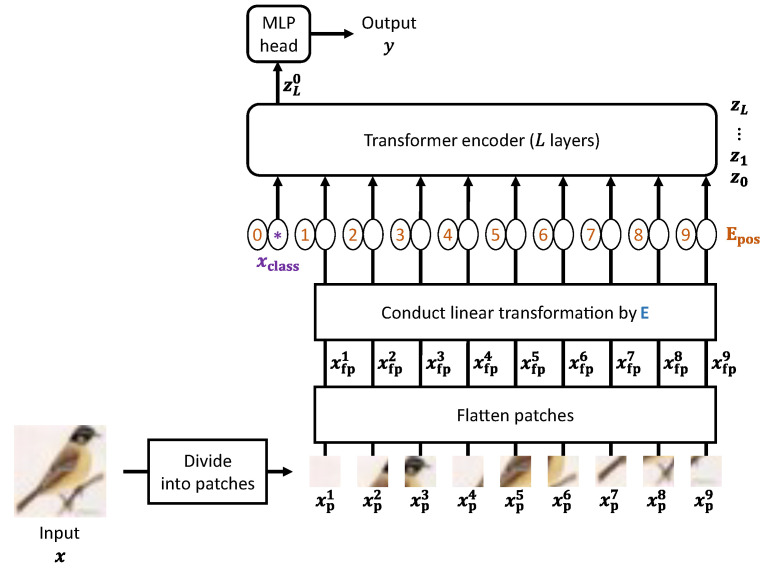
Overview of ViT [21].

Figure 1 shows an overview of ViT. ViT receives an input image $x\in R^{H\times W\times C}$ and outputs a prediction class *y* for the image. Here, *H*, *W*, and *C* denote the height, width, and number of channels of the input image, respectively. First, ViT divides x into patches $x_{p}^{\alpha }\in R^{P\times P\times C}$, where *P* is the patch size, and α∈{1,2,⋯,N}. *N* represents the number of $x_{p}^{\alpha }$. Here, we define a patch set $x_{p}\in R^{N\times P\times P\times C}$ as

(1) $$x_{p}=\left(x_{p}^{1}\;\;\;x_{p}^{2}\;\;\;\cdots \;\;\;x_{p}^{N}\right).$$

Each patch is then flattened to generate $x_{\mathrm{fp}}^{\alpha }\in R^{P^{2}C}$ with a single dimension. We call $x_{\mathrm{fp}}^{\alpha }$ a flattened patch. $x_{\mathrm{fp}}^{\alpha }$ is linearly transformed into a vector with *D* dimensions using a matrix $E\in R^{(P^{2}C)\times D}$, where *D* is the number of vector dimensions received by the transformer encoder. Further, a class token $x_{\mathrm{class}}\in R^{D}$ is located at the head of the sequence of vectors. The position information $E_{\mathrm{pos}}\in R^{(N+1)\times D}$ is then embedded into the sequence of vectors so as to generate a matrix $z_{0}\in R^{(N+1)\times D}$ that is input to the transformer encoder. In summary, $z_{0}$ is represented as

(2) $$z_{0}={\left(x_{\mathrm{class}}\;\;\;x_{\mathrm{fp}}^{1}E\;\;\;x_{\mathrm{fp}}^{2}E\;\;\;\cdots \;\;\;x_{\mathrm{fp}}^{N}E\right)}^{T}+E_{\mathrm{pos}}.$$

The transformer encoder contains *L* layers, and each layer consists of multi-head self-attention (MSA), multi-layer perceptron (MLP), and layer normalization (LN). The transformer encoder receives and transforms $z_{0}$ as

(3) $$\begin{array}{c} \left\{\begin{array}{c} z_{l}=\mathrm{MLP}\left(\mathrm{LN}\left(z_{l}^{\prime }\right)\right)+z_{l}^{\prime },\;\;\;l\in \left\{1,2,\cdots ,L\right\},\\ z_{l}^{\prime }=\mathrm{MSA}\left(\mathrm{LN}\left(z_{l-1}\right)\right)+z_{l-1}. \end{array}\right. \end{array}$$

Here, $z_{l}$ denotes the output from the *l*-th layer; thus, $z_{L}$ means the output from the final layer of the transformer encoder. Finally, *y* is derived from $z_{L}^{0}$, which is the head row in $z_{L}$:

(4) $$y=\mathrm{LN}(z_{L}^{0}).$$

From Equation (2), it is clear that the β-th pixel in every flattened patch is transformed by the β-th row in E, where $\beta \in \{1,2,\cdots ,P^{2}C\}$. On the other hand, the (α+1)-th row in $E_{\mathrm{pos}}$ is added to $x_{\mathrm{fp}}^{\alpha }E$. By focusing on these properties of ViT, the authors previously proposed a model-encryption method that corresponds to EtC images [17]. Our previous method can classify EtC images without any degradation to the classification accuracy. We outline our previous method in the following section.

### 2.2. Previous Classification Method for EtC Images through Encrypted ViT Model

**Figure 2:**
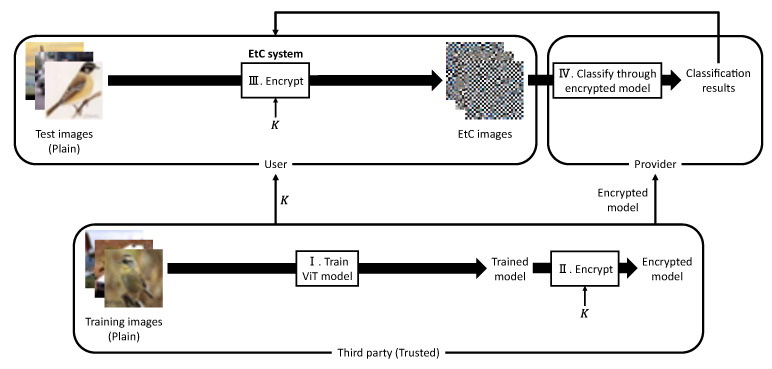
Block diagram of the previous method [17].

This section describes our previous method that enables us to protect both a trained ViT model and test images while preserving high classification accuracy [17]. The test images can be efficiently compressed using lossless image compression standards. Figure 2 shows a block diagram of the previous method. Note that any images used in this method have RGB color channels. In this method, we assume a model in which there exist a single user, provider, and trusted third party. First, the trusted third party trains a ViT model with training images in the plane domain.

The parameters E and $E_{\mathrm{pos}}$ in the trained ViT model are then transformed by a key set $K=\{K_{1},K_{2},K_{3},K_{4},K_{5}\}$ to encrypt the trained model. This process is called model encryption. The trusted third party transmits the encrypted model to the provider and the key set *K* to the user. The user encrypts test images using the EtC system [18] with *K*. This process will hereafter be called image encryption. The EtC images are subsequently transmitted to the provider. The provider obtains the classification results for the EtC images through the encrypted model and finally sends the classification results back to the user. The image and model-encryption procedures are detailed in Section 3.2 and Section 3.3, respectively.

In this system, the user transmits the EtC images to the provider to obtain the classification results. Thus, the encrypted model is not disclosed to anyone outside the provider. This means that no one outside the provider can access and manipulate the encrypted model. The user, therefore, cannot decrypt the encrypted model despite having *K*. On the other hand, it is difficult to decrypt the EtC images without using *K*. The trusted third party does not provide *K* but the encrypted model itself to the provider, so the provider cannot decrypt the EtC images and expose the image content. Therefore, this system prevents unauthorized persons/organizations from obtaining plain test images and a plain model.

Using the previous method, we can obtain a suitable model for EtC images by encrypting the trained model. Accordingly, the classification results for EtC images through an encrypted model are identical to those for plain test images through a plain model. Furthermore, EtC images are expected to have a high compression performance since the encryption system employs a block-wise transformation. The previous method demonstrated that JPEG-LS compression [23] could significantly reduce the data amount of EtC images.

In contrast, JPEG is the most popular standard for lossy image compression. Thus, in this paper, we examine the effects of JPEG compression for EtC images on the classification accuracy and further assess the tradeoff between the accuracy and compression performance. To the best of our knowledge, this is the first study on image classification that maintains high classification accuracy against JPEG compression.

## 3. Evaluation of JPEG-Compression Effects on the Classification Results

This paper extends the previous method [17] to verify the effects of JPEG compression for EtC images on the classification results. This section first outlines evaluation schemes to investigate the JPEG-compression effects and then details the image and model-encryption procedure. Finally, we describe the evaluation metrics in our experiments.

### 3.1. Overview

**Figure 3:**
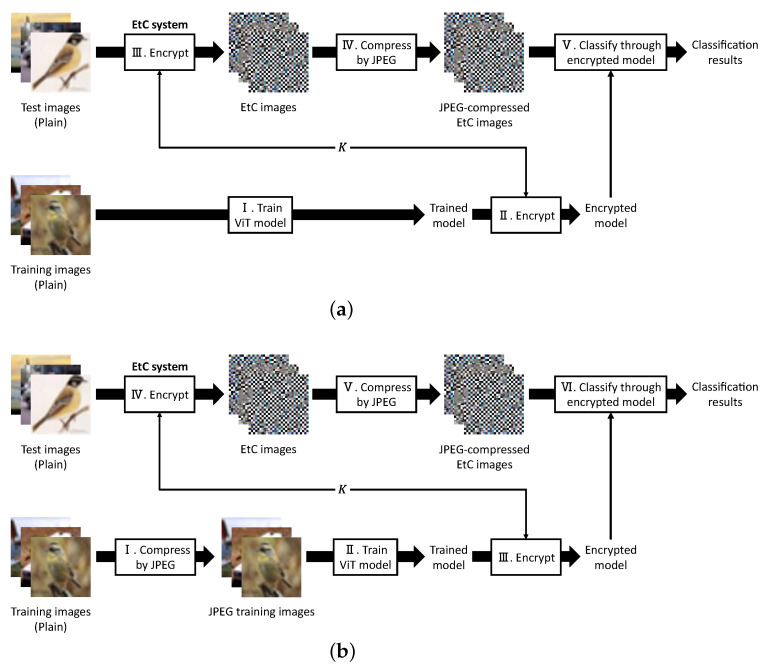
Classification flow of evaluation schemes. (**a**) Classification flow for JPEG-compressed EtC images using the encrypted model trained with plain training images (evaluation scheme A, hereafter). (**b**) Classification flow for JPEG-compressed EtC images using the encrypted model trained with JPEG training images (evaluation scheme B, hereafter).

Figure 3 illustrates the flows of our evaluation schemes. We prepared two types of schemes to elaborately examine the effects of JPEG compression. Hereafter, the schemes shown in Figure 3a,b will be called evaluation schemes A and B, respectively. Note that all images used in this paper have RGB color channels.

First, a ViT model is trained by using plain training images in scheme A. In scheme B, the plain training-images are preliminarily compressed by JPEG, and the ViT model is trained by using the compressed images (JPEG training images, hereafter). The flow after model training is the same between the two evaluation schemes. A trusted third-party encrypts the trained model with a key set $K=\{K_{1},K_{2},K_{3},K_{4},K_{5}\}$, and *K* and the encrypted model are transmitted to a user and a provider, respectively. The user encrypts test images using the EtC system [18] and compresses the EtC images by JPEG. The JPEG-compressed EtC images are then sent to the provider to be classified. The provider classifies each JPEG-compressed EtC image through the encrypted model and finally returns the classification results to the user.

In scheme A, test images encrypted by the EtC system are compressed by JPEG. Thus, we verify the compression effects for test images through comparison with our previous method [17]. In comparison, both training and test images are compressed by JPEG in scheme B. Through a comparison between schemes A and B, we examine the compression effects for training images on the classification of JPEG-compressed test images.

### 3.2. Image Encryption

**Figure 4:**
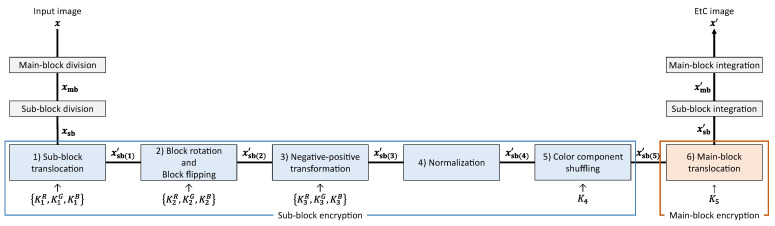
Image-encryption procedure.

Figure 4 shows an image-encryption procedure. This encryption algorithm is an extension of the block-based image-encryption method [18], which is one of the EtC systems. We preliminarily prepare a key set $K=\{K_{1},K_{2},K_{3},K_{4},K_{5}\}$ so as to encrypt an input image. Note that $K_{1}$, $K_{2}$, and $K_{3}$ are key sets consisting of three keys $\left\{K_{q}^{R},K_{q}^{G},K_{q}^{B}\right\}$(q=1,2,3), and $K_{4}$ and $K_{5}$ represent single keys. The image-encryption procedure is described as follows.

Step i-1: Divide an input image into main blocks, and further divide each main block into sub blocks.
Step i-2: Translocate sub blocks within each main block using $K_{1}$.
Step i-3: Rotate and flip each sub block using $K_{2}$.
Step i-4: Apply a negative–positive transformation to each sub block using $K_{3}$.
Step i-5: Normalize all pixels.
Step i-6: Shuffle the R, G, and B components in each sub block using $K_{4}$.
Step i-7: Translocate main blocks using $K_{5}$.
Step i-8: Integrate all of the sub and main blocks.

**Figure 5:**
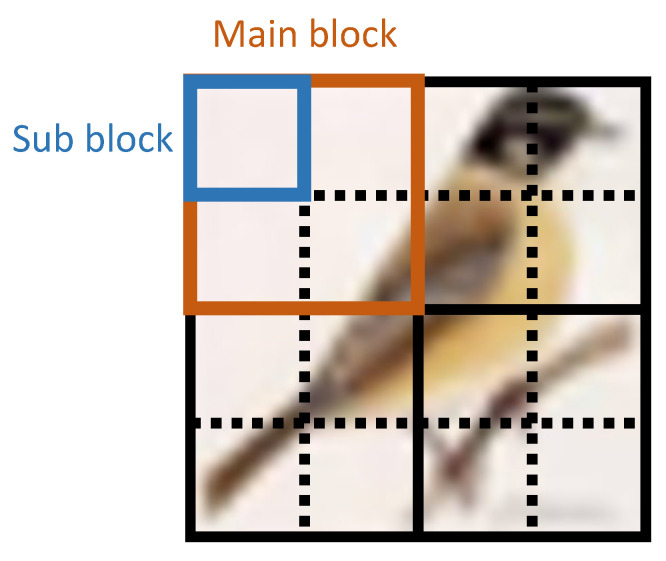
Main-block and sub-block divisions.

In Step i-1, the input image is divided into main and sub blocks as shown in Figure 5. We call Steps i-2 to i-6 sub-block encryption and Step i-7 main-block encryption.

Sub-block encryption includes five operations. Each operation, except normalization, is a sub-block-wise transformation in each main block, and $K_{1}$, $K_{2}$, $K_{3}$, and $K_{4}$ are shared among all the main blocks. $K_{q}$(q=1,2,3) consist of three single keys $K_{q}^{R}$, $K_{q}^{G}$, and $K_{q}^{B}$ corresponding to the R, G, and B components, respectively. Thus, each component can be transformed independently when $K_{q}^{R}$, $K_{q}^{G}$, and $K_{q}^{B}$ are different from each other. In contrast, all the components are transformed commonly when the three keys are identical.

The former is called independent transformation, and the latter is called common transformation in this paper. The main-block encryption consists of a single operation, where the main blocks are translocated. Since $K_{5}$ for the main-block encryption is not a key set but a single key, the R, G, and B components should be translocated commonly. The encryption algorithm transforms an input image while preserving the pixel-to-pixel correlation in each sub block, and so the encrypted image is expected to be highly compressed.

Before we detail the sub-block and main-block encryptions, symbols are preliminarily defined as follows.

- *H* and *W*: the height and width of an image.
- $x\in {\left\{0,1,\cdots ,255\right\}}^{H\times W\times 3}$: an input image.
- $S_{\mathrm{mb}}$ and $S_{\mathrm{sb}}$: the main-block and sub-block sizes.
- $N_{\mathrm{mb}}$: the number of main blocks.
- $N_{\mathrm{sb}}$: the number of sub blocks within each main block.
- $x_{\mathrm{mb}}\in {\left\{0,1,\cdots ,255\right\}}^{N_{\mathrm{mb}}\times S_{\mathrm{mb}}\times S_{\mathrm{mb}}\times 3}$: an image after main-block division, called a main-block image.
- $x_{\mathrm{sb}}\in {\left\{0,1,\cdots ,255\right\}}^{N_{\mathrm{mb}}\times N_{\mathrm{sb}}\times S_{\mathrm{sb}}\times S_{\mathrm{sb}}\times 3}$: an image after sub-block division, called a sub-block image.
- ${x^{\prime }}_{\mathrm{sb}(\gamma )}\in {\left\{0,1,\cdots ,255\right\}}^{N_{\mathrm{mb}}\times N_{\mathrm{sb}}\times S_{\mathrm{sb}}\times S_{\mathrm{sb}}\times 3}$: an image after the γ-th operation in sub-block encryption, where γ∈{1,2,3,4,5}.
- ${x^{\prime }}_{\mathrm{sb}}\in {\left\{0,1,\cdots ,255\right\}}^{N_{\mathrm{mb}}\times N_{\mathrm{sb}}\times S_{\mathrm{sb}}\times S_{\mathrm{sb}}\times 3}$: an image after main-block encryption.
- ${x^{\prime }}_{\mathrm{mb}}\in {\left\{0,1,\cdots ,255\right\}}^{N_{\mathrm{mb}}\times S_{\mathrm{mb}}\times S_{\mathrm{mb}}\times 3}$: an image after sub-block integration.
- $x^{\prime }\in {\left\{0,1,\cdots ,255\right\}}^{H\times W\times 3}$: an image after main-block integration, i.e., an EtC image.
- $x_{\mathrm{sb}}\left(m,s,h,w,c\right)$, $x_{\mathrm{sb}(\gamma )}^{\prime }\left(m,s,h,w,c\right)$, and $x_{\mathrm{sb}}^{\prime }\left(m,s,h,w,c\right)$: pixel values in $x_{\mathrm{sb}}$, ${x^{\prime }}_{\mathrm{sb}(\gamma )}$, and ${x^{\prime }}_{\mathrm{sb}}$, respectively.
  - $m\in \{1,2,\cdots ,N_{\mathrm{mb}}\}$: a main-block number.
  - $s\in \{1,2,\cdots ,N_{\mathrm{sb}}\}$: a sub-block number in the *m*-th main block.
  - $h\in \{1,2,\cdots ,S_{\mathrm{sb}}\}$: a position in the height direction in the *s*-th sub block.
  - $w\in \{1,2,\cdots ,S_{\mathrm{sb}}\}$: a position in the width direction in the *s*-th sub block.
  - c∈{1,2,3}: a color-channel number.

#### 3.2.1. Sub-Block Translocation

We first translocate sub blocks within each main block by using $K_{1}$. Vectors $v^{i}$(i∈{1,2,3}) are generated by $K_{1}^{R}$, $K_{1}^{G}$, and $K_{1}^{B}$, respectively. Each vector $v^{i}$ is represented as

(5) $$v^{i}=\left(v_{1}^{i},v_{2}^{i},\cdots ,v_{j}^{i},\cdots ,v_{\hat{j}}^{i},\cdots ,v_{N_{\mathrm{sb}}}^{i}\right),$$

where $v_{j}^{i},v_{\hat{j}}^{i}\in \left\{1,2,\cdots ,N_{\mathrm{sb}}\right\}$, and $v_{j}^{i}\neq v_{\hat{j}}^{i}$ if $j\neq \hat{j}$. The second dimension of $x_{\mathrm{sb}}$ denotes a sub-block number; thus, the sub blocks are translocated by replacing their numbers with $v^{i}$:

(6) $$x_{\mathrm{sb}(1)}^{\prime }\left(m,j,h,w,i\right)=x_{\mathrm{sb}}\left(m,v_{j}^{i},h,w,i\right).$$

#### 3.2.2. Block Rotation and Block Flipping

**Figure 6:**
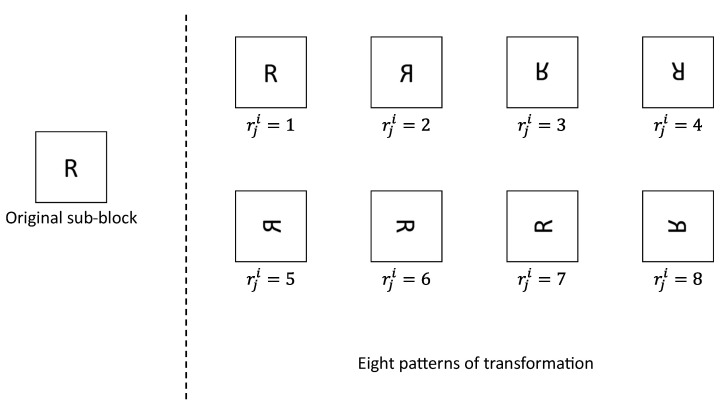
Transformation patterns in block rotation and block flipping.

Next, we rotate and flip each sub block using $K_{2}$. As shown in Figure 6, there are eight transformation patterns for each sub block. Three vectors $r^{i}$(i∈{1,2,3}) are derived from $K_{2}^{R}$, $K_{2}^{G}$, and $K_{2}^{B}$, respectively. Each vector $r^{i}$ is denoted by

(7) $$r^{i}=\left(r_{1}^{i},r_{2}^{i},\cdots ,r_{j}^{i},\cdots ,r_{N_{\mathrm{sb}}}^{i}\right),$$

where $r_{j}^{i}\in \left\{1,2,\cdots ,8\right\}$. The third and fourth dimensions of ${x^{\prime }}_{\mathrm{sb}(1)}$ represent the position in the height and width directions in each sub block, respectively. Therefore, each sub block is rotated and flipped by translocating pixels within the sub block depending on $r^{i}$:

(8) $$x_{\mathrm{sb}(2)}^{\prime }\left(m,j,h,w,i\right)=\left\{\begin{array}{cc} x_{\mathrm{sb}(1)}^{\prime }\left(m,j,h,w,i\right) & \left(r_{j}^{i}=1\right)\\ x_{\mathrm{sb}(1)}^{\prime }\left(m,j,h,R_{w},i\right) & \left(r_{j}^{i}=2\right)\\ x_{\mathrm{sb}(1)}^{\prime }\left(m,j,R_{h},w,i\right) & \left(r_{j}^{i}=3\right)\\ x_{\mathrm{sb}(1)}^{\prime }\left(m,j,R_{h},R_{w},i\right) & \left(r_{j}^{i}=4\right)\\ x_{\mathrm{sb}(1)}^{\prime }\left(m,j,w,h,i\right) & \left(r_{j}^{i}=5\right)\\ x_{\mathrm{sb}(1)}^{\prime }\left(m,j,w,R_{h},i\right) & \left(r_{j}^{i}=6\right)\\ x_{\mathrm{sb}(1)}^{\prime }\left(m,j,R_{w},h,i\right) & \left(r_{j}^{i}=7\right)\\ x_{\mathrm{sb}(1)}^{\prime }\left(m,j,R_{w},R_{h},i\right) & (r_{j}^{i}=8), \end{array}\right.$$

where $R_{h}=S_{\mathrm{sb}}-h+1$, and $R_{w}=S_{\mathrm{sb}}-w+1$.

#### 3.2.3. Negative–Positive Transformation

We then apply a negative–positive transformation to each sub block with $K_{3}$. Vectors $n^{i}$(i∈{1,2,3}) are generated using $K_{3}^{R}$, $K_{3}^{G}$, and $K_{3}^{B}$ and given by

(9) $$n^{i}=\left(n_{1}^{i},n_{2}^{i},\cdots ,n_{j}^{i},\cdots ,n_{N_{\mathrm{sb}}}^{i}\right),$$

where $n_{j}^{i}\in \left\{1,2\right\}$. The negative–positive transformation is conducted on the basis of $n^{i}$:

(10) $$x_{\mathrm{sb}(3)}^{\prime }\left(m,j,h,w,i\right)=\left\{\begin{array}{cc} x_{\mathrm{sb}(2)}^{\prime }\left(m,j,h,w,i\right) & \left(n_{j}^{i}=1\right)\\ 255-x_{\mathrm{sb}(2)}^{\prime }\left(m,j,h,w,i\right) & (n_{j}^{i}=2). \end{array}\right.$$

#### 3.2.4. Normalization

All pixels in ${x^{\prime }}_{\mathrm{sb}(3)}$ should be normalized as

(11) $$x_{\mathrm{sb}(4)}^{\prime }\left(m,s,h,w,c\right)=\frac{x_{\mathrm{sb}(3)}^{\prime }\left(m,s,h,w,c\right)-255/2}{S},$$

where *S* is an arbitrary constant, while S=255/2 in this paper. In the case of $n_{j}^{i}=1$ in Equation (10), $x_{\mathrm{sb}(4)}^{\prime }\left(m,s,h,w,c\right)$ can be expressed as

(12) $$\begin{array}{ccc} x_{\mathrm{sb}(4)}^{\prime }\left(m,j,h,w,i\right) & = & \frac{x_{\mathrm{sb}(3)}^{\prime }\left(m,j,h,w,i\right)-255/2}{S}\\ & = & \frac{x_{\mathrm{sb}(2)}^{\prime }\left(m,j,h,w,i\right)-255/2}{S}. \end{array}$$

Otherwise, $x_{\mathrm{sb}(4)}^{\prime }\left(m,s,h,w,c\right)$ is given by

(13) $$\begin{array}{ccc} x_{\mathrm{sb}(4)}^{\prime }\left(m,j,h,w,i\right) & = & \frac{x_{\mathrm{sb}(3)}^{\prime }\left(m,j,h,w,i\right)-255/2}{S}\\ & = & \frac{(255-x_{\mathrm{sb}(2)}^{\prime }\left(m,j,h,w,i\right))-255/2}{S}\\ & = & -\frac{x_{\mathrm{sb}(2)}^{\prime }\left(m,j,h,w,i\right)-255/2}{S}. \end{array}$$

From Equations (12) and (13), it is clear that the negative–positive transformation with normalization can be regarded as an operation of retaining or flipping the sign of each pixel value. This property prevents a model encryption algorithm from being complex. We detail the algorithm in Section 3.3.3.

#### 3.2.5. Color Component Shuffling

We then shuffle the R, G, and B components in each sub block using $K_{4}$. A vector a is derived from $K_{4}$ and represented as

(14) $$\begin{array}{c} a=(a_{1},a_{2},\cdots ,a_{j},\cdots ,a_{N_{\mathrm{sb}}}), \end{array}$$

where $a_{j}\in \left\{1,2,\cdots ,6\right\}$. The fifth dimension of ${x^{\prime }}_{\mathrm{sb}(4)}$ denotes a color-channel number; this operation swaps pixel values among the color components according to a:

(15) $$x_{\mathrm{sb}(5)}^{\prime }\left(m,j,h,w,1\right)=\left\{\begin{array}{cc} x_{\mathrm{sb}(4)}^{\prime }\left(m,j,h,w,1\right) & \left(a_{j}=1\;\mathrm{or}\;2\right)\\ x_{\mathrm{sb}(4)}^{\prime }\left(m,j,h,w,2\right) & \left(a_{j}=3\;\mathrm{or}\;4\right)\\ x_{\mathrm{sb}(4)}^{\prime }\left(m,j,h,w,3\right) & (a_{j}=5\;\mathrm{or}\;6), \end{array}\right.$$

(16) $$x_{\mathrm{sb}(5)}^{\prime }\left(m,j,h,w,2\right)=\left\{\begin{array}{cc} x_{\mathrm{sb}(4)}^{\prime }\left(m,j,h,w,1\right) & \left(a_{j}=3\;\mathrm{or}\;5\right)\\ x_{\mathrm{sb}(4)}^{\prime }\left(m,j,h,w,2\right) & \left(a_{j}=1\;\mathrm{or}\;6\right)\\ x_{\mathrm{sb}(4)}^{\prime }\left(m,j,h,w,3\right) & (a_{j}=2\;\mathrm{or}\;4), \end{array}\right.$$

and

(17) $$x_{\mathrm{sb}(5)}^{\prime }\left(m,j,h,w,3\right)=\left\{\begin{array}{cc} x_{\mathrm{sb}(4)}^{\prime }\left(m,j,h,w,1\right) & \left(a_{j}=4\;\mathrm{or}\;6\right)\\ x_{\mathrm{sb}(4)}^{\prime }\left(m,j,h,w,2\right) & \left(a_{j}=2\;\mathrm{or}\;5\right)\\ x_{\mathrm{sb}(4)}^{\prime }\left(m,j,h,w,3\right) & (a_{j}=1\;\mathrm{or}\;3). \end{array}\right.$$

#### 3.2.6. Main-Block Translocation

Finally, the main blocks are translocated with $K_{5}$. A vector k obtained by $K_{5}$ is given by

(18) $$k=(k_{1},k_{2},\cdots ,k_{t},\cdots ,k_{\hat{t}},\cdots ,k_{N_{\mathrm{mb}}}),$$

where $k_{t},k_{\hat{t}}\in \left\{1,2,\cdots ,N_{\mathrm{mb}}\right\}$, and $k_{t}\neq k_{\hat{t}}$ if $t\neq \hat{t}$. The first dimension of ${x^{\prime }}_{\mathrm{sb}(5)}$ represents a main-block number, so we translocate the main blocks by replacing their numbers with k:

(19) $$x_{\mathrm{sb}}^{\prime }\left(t,s,h,w,c\right)=x_{\mathrm{sb}(5)}^{\prime }\left(k_{t},s,h,w,c\right).$$

### 3.3. Model Encryption

This section describes the model-encryption procedure. While image encryption can protect visual information, it seriously deteriorates the classification accuracy. The model encryption in this paper not only cancels out the effects but also prevents unauthorized accesses to a trained ViT model by encryption.

We assume that the patch size *P* in ViT is the same as the main-block size $S_{\mathrm{mb}}$ in the image encryption and that the number of patches *N* is equal to the number of main blocks $N_{\mathrm{mb}}$. The patch set $x_{p}$ has N×P×P×3 dimensions, and the main-block image $x_{\mathrm{mb}}$ has $N_{\mathrm{mb}}\times S_{\mathrm{mb}}\times S_{\mathrm{mb}}\times 3$ dimensions—namely, $x_{p}$ and $x_{\mathrm{mb}}$ are identical. Here, we define both $x_{\mathrm{mb}}^{\alpha }\in R^{S_{\mathrm{mb}}\times S_{\mathrm{mb}}\times 3}$ and $x_{\mathrm{sb}}^{\alpha }\in R^{N_{\mathrm{sb}}\times S_{\mathrm{sb}}\times S_{\mathrm{sb}}\times 3}$ as a single main block, respectively. Note that α∈{1,2,⋯,N}, and *N* is equal to $N_{\mathrm{mb}}$, and so α is an index denoting the main-block number. $x_{\mathrm{mb}}^{\alpha }$ is a part of $x_{\mathrm{mb}}$ without sub-block division, while $x_{\mathrm{sb}}^{\alpha }$ is a part of $x_{\mathrm{mb}}$ with sub-block division. They are represented as

(20) $$x_{\mathrm{mb}}=\left(x_{\mathrm{mb}}^{1}\;\;\;x_{\mathrm{mb}}^{2}\;\;\;\cdots \;\;\;x_{\mathrm{mb}}^{N_{\mathrm{mb}}}\right),$$

(21) $$x_{\mathrm{sb}}=\left(x_{\mathrm{sb}}^{1}\;\;\;x_{\mathrm{sb}}^{2}\;\;\;\cdots \;\;\;x_{\mathrm{sb}}^{N_{\mathrm{mb}}}\right).$$

$x_{p}$ and $x_{\mathrm{mb}}$ are identical, so the patch $x_{p}^{\alpha }$ and the main block $x_{\mathrm{mb}}^{\alpha }$ are treated as one and the same. Therefore, $x_{\mathrm{fp}}^{\alpha }$ obtained by flattening $x_{p}^{\alpha }$ is also derived from flattening $x_{\mathrm{mb}}^{\alpha }$. Hereafter, *P* and *N* will be denoted as $S_{\mathrm{mb}}$ and $N_{\mathrm{mb}}$, respectively, for the sake of consistency.

**Figure 7:**
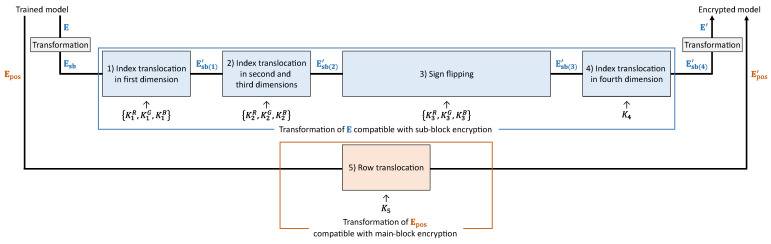
Model-encryption procedure.

Figure 7 illustrates a model-encryption procedure. One of the purposes of model encryption is to ensure that the classification results are never affected by image encryption. Thus, we transform the parameters E and $E_{\mathrm{pos}}$ in the trained model with the key set *K*, which is the same as for the image encryption. Each operation in the model encryption is compatible with each operation in the image encryption. The model-encryption procedure is described as follows.

Step m-1: Transform E to obtain $E_{\mathrm{sb}}\in R^{N_{\mathrm{sb}}\times S_{\mathrm{sb}}\times S_{\mathrm{sb}}\times 3\times D}$.
Step m-2: Translocate indices in the first dimension of $E_{\mathrm{sb}}$ using $K_{1}$.
Step m-3: Translocate indices in the second and third dimensions of $E_{\mathrm{sb}}$ using $K_{2}$.
Step m-4: Flip or retain the signs of the elements in $E_{\mathrm{sb}}$ using $K_{3}$.
Step m-5: Translocate indices in the fourth dimension of $E_{\mathrm{sb}}$ using $K_{4}$.
Step m-6: Transform $E_{\mathrm{sb}}$ into the original dimension of E to derive $E^{\prime }\in R^{(3\cdot S_{\mathrm{mb}}\cdot S_{\mathrm{mb}})\times D}$.
Step m-7: Translocate rows in $E_{\mathrm{pos}}$ using $K_{5}$ to obtain $E_{\mathrm{pos}}^{\prime }\in R^{(N_{\mathrm{mb}}+1)\times D}$.

**Figure 8:**
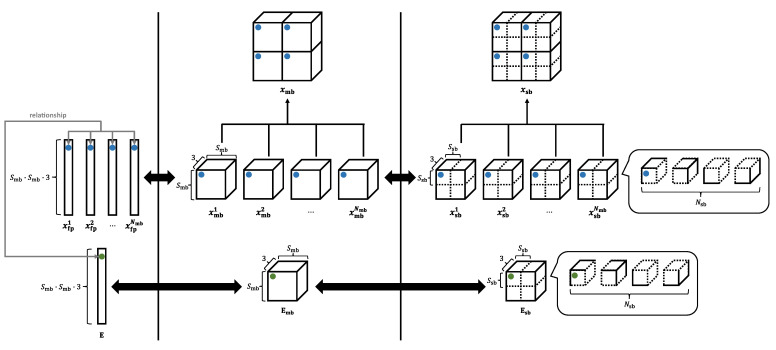
Relationship between divided the image and ViT parameter E. Blue dots represent single pixels in the segmented image, and green dots represent single rows in E corresponding to blue dots.

Figure 8 illustrates the relationship between a divided image and E. We transform E to $E_{\mathrm{mb}}\in R^{S_{\mathrm{mb}}\times S_{\mathrm{mb}}\times 3\times D}$ and then obtain $E_{\mathrm{sb}}$ in Step m-1. This step allows E to be encrypted directly by using the vectors for the sub-block encryption.

As mentioned in Section 2.1, E and $E_{\mathrm{pos}}$ correspond to $x_{\mathrm{fp}}^{\alpha }$ and $x_{\mathrm{fp}}^{\alpha }E$, respectively. Each operation in the image encryption generally sacrifices their correspondence. Accordingly, the common image-encryption methods significantly degrade the classification accuracy. In contrast, an image-encryption method based on the EtC system is compatible with each parameter of ViT. Taking advantage of this compatibility, we proposed a model-encryption method for ViT without any degradation to the classification accuracy caused by image encryption [17]. Our previous method demonstrated that the classification accuracy was never affected by encryption [25].

We detail each operation in the model encryption below. Hereafter, $\;E_{\mathrm{sb}(\delta )}^{\prime }\;\in \;R^{N_{\mathrm{sb}}\times S_{\mathrm{sb}}\times S_{\mathrm{sb}}\times 3\times D}\;$, where δ∈{1,2,3,4}, represents a parameter after the δ-th operation to E. Further, $E_{\mathrm{sb}}\left(s,h,w,c,d\right)$ and $E_{\mathrm{sb}(\delta )}^{\prime }\left(s,h,w,c,d\right)$, where d∈{1,2,⋯,D}, denote the elements of $E_{\mathrm{sb}}$ and $E_{\mathrm{sb}(\delta )}^{\prime }$, respectively.

#### 3.3.1. Index Translocation in the First Dimension

We first translocate indices in the first dimension of $E_{\mathrm{sb}}$. On the basis of Equation (6), the sub-block translocation replaces the indices in the second dimension of $x_{\mathrm{sb}}$ with vectors $v^{i}$ derived using $K_{1}$. The second dimension of $x_{\mathrm{sb}}$ corresponds to the first dimension of $E_{\mathrm{sb}}$. Thus, the indices in the first dimension of $E_{\mathrm{sb}}$ should be translocated by replacing them with $v^{i}$:

(22) $$E_{\mathrm{sb}(1)}^{\prime }\left(j,h,w,i,d\right)=E_{\mathrm{sb}}\left(v_{j}^{i},h,w,i,d\right).$$

#### 3.3.2. Index Translocation in the Second and Third Dimensions

Next, we translocate indices in the second and third dimensions of $E_{\mathrm{sb}(1)}^{\prime }$. As shown in Equation (8), the block rotation and block flipping translocates the indices in the third and fourth dimensions of ${x^{\prime }}_{\mathrm{sb}(1)}$ in response to vectors $r^{i}$ derived from $K_{2}$. The third and fourth dimensions of ${x^{\prime }}_{\mathrm{sb}(1)}$ are compatible with the second and third dimensions of $E_{\mathrm{sb}(1)}^{\prime }$, respectively. The indices in the second and third dimensions of $E_{\mathrm{sb}(1)}^{\prime }$ should be translocated accordingly depending on $r^{i}$:

(23) $$E_{\mathrm{sb}(2)}^{\prime }\left(j,h,w,i,d\right)=\left\{\begin{array}{cc} E_{\mathrm{sb}(1)}^{\prime }\left(j,h,w,i,d\right) & \left(r_{j}^{i}=1\right)\\ E_{\mathrm{sb}(1)}^{\prime }\left(j,h,R_{w},i,d\right) & \left(r_{j}^{i}=2\right)\\ E_{\mathrm{sb}(1)}^{\prime }\left(j,R_{h},w,i,d\right) & \left(r_{j}^{i}=3\right)\\ E_{\mathrm{sb}(1)}^{\prime }\left(j,R_{h},R_{w},i,d\right) & \left(r_{j}^{i}=4\right)\\ E_{\mathrm{sb}(1)}^{\prime }\left(j,w,h,i,d\right) & \left(r_{j}^{i}=5\right)\\ E_{\mathrm{sb}(1)}^{\prime }\left(j,w,R_{h},i,d\right) & \left(r_{j}^{i}=6\right)\\ E_{\mathrm{sb}(1)}^{\prime }\left(j,R_{w},h,i,d\right) & \left(r_{j}^{i}=7\right)\\ E_{\mathrm{sb}(1)}^{\prime }\left(j,R_{w},R_{h},i,d\right) & (r_{j}^{i}=8). \end{array}\right.$$

#### 3.3.3. Sign Flipping

Here, we flip signs of the elements in $E_{\mathrm{sb}(2)}^{\prime }$. As described in Section 3.2.4, the negative–positive transformation with normalization is regarded as an operation to flip or retain the signs of the pixel values in ${x^{\prime }}_{\mathrm{sb}(2)}$. We determine whether to flip or retain the signs of the elements in $E_{\mathrm{sb}(2)}^{\prime }$ responding to vectors $n^{i}$ generated using $K_{3}$. $E_{\mathrm{sb}(2)}^{\prime }$ is consequently transformed as

(24) $$E_{\mathrm{sb}(3)}^{\prime }\left(j,h,w,i,d\right)=\left\{\begin{array}{cc} E_{\mathrm{sb}(2)}^{\prime }\left(j,h,w,i,d\right) & \left(n_{j}^{i}=1\right)\\ -E_{\mathrm{sb}(2)}^{\prime }\left(j,h,w,i,d\right) & (n_{j}^{i}=2). \end{array}\right.$$

#### 3.3.4. Index Translocation in Fourth Dimension

We then translocate indices in the fourth dimension of $E_{\mathrm{sb}(3)}^{\prime }$. As shown in Equations (15)–(17), the color component shuffling translocates the indices in the fifth dimension of ${x^{\prime }}_{\mathrm{sb}(4)}$ on the basis of the vector a derived using $K_{4}$. The fifth dimension of ${x^{\prime }}_{\mathrm{sb}(4)}$ corresponds to the fourth dimension of $E_{\mathrm{sb}(3)}^{\prime }$. We, thus, translocate the indices in the fourth dimension of $E_{\mathrm{sb}(3)}^{\prime }$ by using a:

(25) $$E_{\mathrm{sb}(4)}^{\prime }\left(j,h,w,1,d\right)=\left\{\begin{array}{cc} E_{\mathrm{sb}(3)}^{\prime }\left(j,h,w,1,d\right) & \left(a_{j}=1\;\mathrm{or}\;2\right)\\ E_{\mathrm{sb}(3)}^{\prime }\left(j,h,w,2,d\right) & \left(a_{j}=3\;\mathrm{or}\;4\right)\\ E_{\mathrm{sb}(3)}^{\prime }\left(j,h,w,3,d\right) & (a_{j}=5\;\mathrm{or}\;6), \end{array}\right.$$

(26) $$E_{\mathrm{sb}(4)}^{\prime }\left(j,h,w,2,d\right)=\left\{\begin{array}{cc} E_{\mathrm{sb}(3)}^{\prime }\left(j,h,w,1,d\right) & \left(a_{j}=3\;\mathrm{or}\;5\right)\\ E_{\mathrm{sb}(3)}^{\prime }\left(j,h,w,2,d\right) & \left(a_{j}=1\;\mathrm{or}\;6\right)\\ E_{\mathrm{sb}(3)}^{\prime }\left(j,h,w,3,d\right) & (a_{j}=2\;\mathrm{or}\;4), \end{array}\right.$$

and

(27) $$E_{\mathrm{sb}(4)}^{\prime }\left(j,h,w,3,d\right)=\left\{\begin{array}{cc} E_{\mathrm{sb}(3)}^{\prime }\left(j,h,w,1,d\right) & \left(a_{j}=4\;\mathrm{or}\;6\right)\\ E_{\mathrm{sb}(3)}^{\prime }\left(j,h,w,2,d\right) & \left(a_{j}=2\;\mathrm{or}\;5\right)\\ E_{\mathrm{sb}(3)}^{\prime }\left(j,h,w,3,d\right) & (a_{j}=1\;\mathrm{or}\;3). \end{array}\right.$$

#### 3.3.5. Row Translocation

Finally, we translocate rows in $E_{\mathrm{pos}}$. As shown in Equation (19), the main-block translocation replaces the indices in the first dimension of $x_{\mathrm{sb}(5)}^{\prime }$ with vector k obtained by $K_{5}$. Both α and the first dimension of $x_{\mathrm{sb}(5)}^{\prime }$ represent the main-block number, and so the main-block translocation is regarded as an operation to replace α with k. To preserve the relationship between $E_{\mathrm{pos}}$ and $x_{\mathrm{fp}}^{\alpha }E$, the rows in $E_{\mathrm{pos}}$ should accordingly be translocated by using k as

(28) $$E_{\mathrm{pos}}^{\prime }\left(t+1,d\right)=E_{\mathrm{pos}}\left(k_{t}+1,d\right),$$

where $E_{\mathrm{pos}}\left(g,d\right)$ and $E_{\mathrm{pos}}^{\prime }\left(g,d\right)$ denote the elements of $E_{\mathrm{pos}}$ and $E_{\mathrm{pos}}^{\prime }$, respectively. Note that $g\in \{1,2,\cdots ,N_{\mathrm{mb}}+1\}$ is an index corresponding to the dimensions of $E_{\mathrm{pos}}$ and $E_{\mathrm{pos}}^{\prime }$.

### 3.4. Evaluation Metrics

We verified the effectiveness of JPEG compression in terms of compression and classification performance. We calculated the average amount of image data to evaluate the compression performance. In addition, we prepared two metrics to assess the classification performance: the classification accuracy and change rate. In this paper, the change rate provides the percentage of difference between the classification results for plain test images with a plain trained model and those for target images with a target model. For instance, the target images and target model means JPEG-compressed EtC images and an encrypted model, respectively. In the case that the change rate indicates 0%, both classification results are identical.

For scheme A, shown in Figure 3a, we provide five patterns for the quality factor (*Q*): 100, 95, 90, 85, and 80. To compare the effects of JPEG compression, each metric was also calculated for EtC images compressed by linear quantization. In comparison, scheme B, shown in Figure 3b, compressed both training images and EtC images by using JPEG with Q=85. In common with scheme A, the classification accuracy was also calculated for the case of using linear quantization. Hereafter, the EtC images and the training images after the linear quantization are called quantized EtC images and quantized training images, respectively.

## 4. Experiments

In this section, the effects of JPEG compression are examined in terms of classification and compression performance by using the metrics described in Section 3.4.

### 4.1. Experimental Setup

We used the CIFAR-10 dataset with 10 classes in this experiment. This dataset consists of 50,000 training images and 10,000 test images. All image sizes are 32×32 pixels, while we preliminarily resized each image to 224×224 pixels by using the bicubic interpolation method. All training and test images were stored in PPM format.

The ViT model is trained through two phases: pre-training and fine-tuning. In this experiment, we used a pre-trained ViT model using ImageNet-21k with a patch size P=16. We then fine-tuned the pre-trained ViT model by using plain training images for scheme A or JPEG training images for scheme B. In both schemes, the ViT model was fine-tuned with a learning rate of 0.03 and an epoch of 5000.

**Figure 9:**
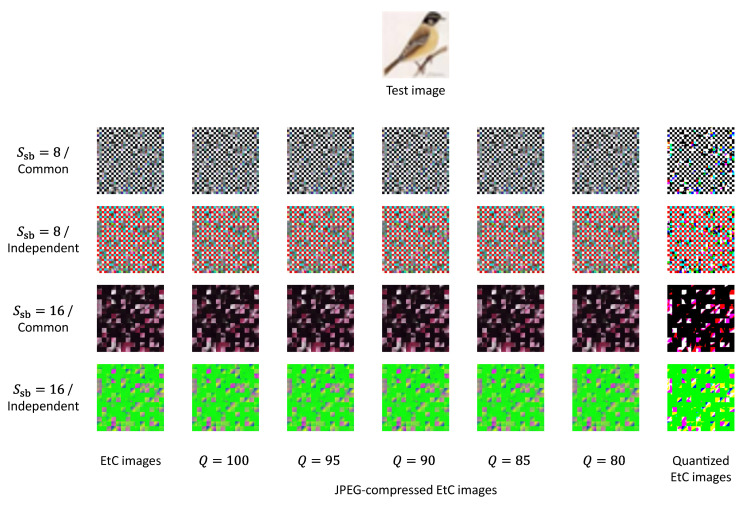
EtC, JPEG-compressed EtC, and quantized EtC images for a single test image.

In the image encryption, the main-block size $S_{\mathrm{mb}}$ was defined as 16, which was the same as *P*, while the sub-block size $S_{\mathrm{sb}}$ was set to 8 or 16. Additionally, as mentioned in Section 3.2, we could choose either the common or independent transformation in regard to color components. Consequently, four types of EtC images were generated for each test image. Figure 9 shows EtC, JPEG-compressed EtC and quantized EtC images for a single test image. Note that we used 4:2:0 downsampling for the JPEG compression.

### 4.2. Experimental Results

Table 1 shows the average amount of data in the JPEG-compressed EtC images and the quantized EtC images. This table also includes the average amount of data in the EtC images without compression and in the plain test images with and without compression. After the linear quantization, pixel values of each color component are represented by a single bit, and so the average amount of image data is 3 bpp. This table indicates that JPEG compression with Q≤95 reduced a larger amount of data than linear quantization. We also found that the JPEG-compressed EtC images with $S_{\mathrm{sb}}=16$ and common transformation had an analogous amount of data to the plain test images with JPEG compression at each value of *Q*.

Table 2 summarizes the classification accuracy and change rate for scheme A. For comparison, this table also gives the results for the quantized EtC images through the encrypted model and for the EtC images without compression through the encrypted model. Note that the latter results could be obtained by our previous method [17]. This table also provides the results for the plain test images with and without compression through the plain model. The change rate is calculated on the basis of the classification results for the plain test images without compression through the plain model.

With each value of *Q*, the classification accuracy and change rate for any encryption pattern were nearly equal to those obtained by using the plain test images and model. It is also clear that JPEG compression for the EtC images preserved a significantly high classification accuracy with a low change rate in any case, while the linear quantization sacrificed the accuracy in return for data reduction. For scheme A, the lowest classification accuracy and highest change rate were obtained in the case of Q=80, $S_{\mathrm{sb}}=8$, and independent transformation. Even with this pattern, the classification accuracy was still 97.67%, and the change rate was still low at 1.94%.

Table 3 shows the classification accuracy for scheme B with Q=85. Here, the model was trained with JPEG training images. In this table, we include the results for the plain test images with JPEG compression through the plain model. For further comparison, this table also includes the results obtained by using linear quantization. In this case, the model was trained with quantized training images. As shown in this table, JPEG compression for both the training images and the EtC images hardly degraded the classification accuracy, while the linear quantization still substantially decreased the accuracy.

Comparing scheme B and scheme A with Q=85 in Table 2, the classification accuracy for the JPEG-compressed EtC images was slightly improved by using the encrypted model trained with the JPEG training images. Accordingly, the results for schemes A and B show that JPEG compression for training images was comparatively effective in improving the classification accuracy for JPEG-compressed EtC images.

### 4.3. Discussion

**Figure 10:**
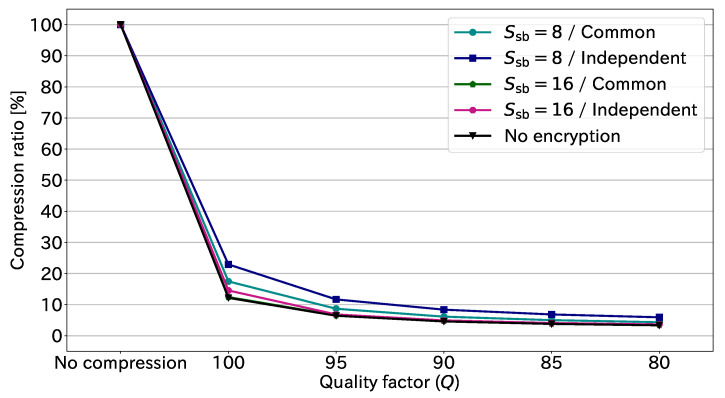
Compression ratio at each quality factor.

Here, we discuss the effects of JPEG compression for EtC images. Figure 10 illustrates the compression ratio at each quality factor. This figure is derived from the results in Table 1. The compression ratio is given by

(29) $$\mathrm{Compression}\;\mathrm{ratio}\;\left[\%\right]=\frac{\mathrm{Average}\;\mathrm{amount}\;\mathrm{of}\;\mathrm{compressed}\;\text{EtC-image}\;\mathrm{data}\;\left[\mathrm{bpp}\right]}{\mathrm{Average}\;\mathrm{amount}\;\mathrm{of}\;\text{EtC-image}\;\mathrm{data}\;\left[\mathrm{bpp}\right]}\times 100.$$

Note that the amount of uncompressed EtC-image data is constantly 24.00 bpp. As shown in Figure 10, the non-encrypted images, i.e., original images, had a comparable performance to the EtC images with $S_{\mathrm{sb}}=16$ and common transformation. This means that the suitable conditions for the EtC system do not affect the compression performance. The figure also shows that JPEG compression could reduce the data amount 75–90% at the highest quality factor, Q=100. Further, the data amount decreased by more than 90% in the case of Q≤90. These results demonstrate that JPEG compression can significantly reduce the amount of EtC-image data.

**Figure 11:**
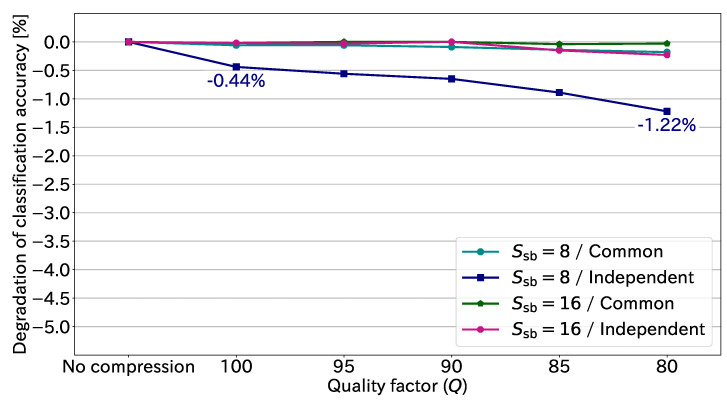
Degradation in classification accuracy at each quality factor.

On the basis of Table 2, we show the degradation in classification accuracy caused by JPEG compression in Figure 11. The negative sign indicates degradation. The maximum degradation in this figure was 1.22% in the case of the independent transformation with $S_{\mathrm{sb}}=8$ and Q=80. Thus, JPEG compression in practical use causes little degradation to the classification accuracy. We can conclude that JPEG compression is effective in drastically reducing the amount of EtC-image data while preserving high classification accuracy.

**Figure 12:**
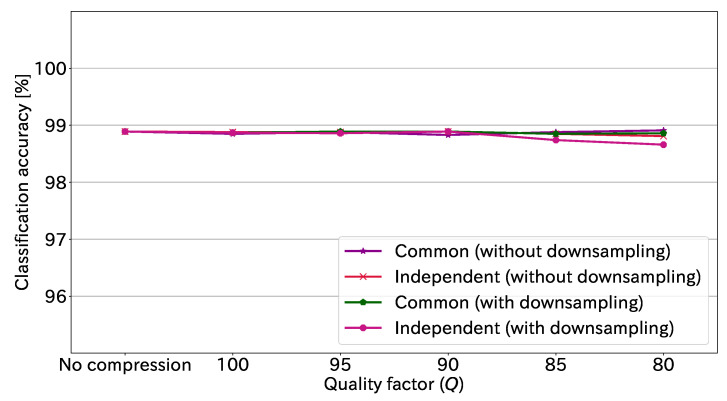
Classification accuracy at each quality factor with and without downsampling ($S_{\mathrm{sb}}=16$).

JPEG compression has an option to not downsample the chrominance component. Figure 12 shows the classification accuracy at each quality factor with and without downsampling. Note that $S_{\mathrm{sb}}$ is 16 in this figure. We confirmed that the classification accuracies with and without downsampling had similar trends.

We employed the EtC system on the premise of applying JPEG compression. The main-block size in the EtC system was the same as the patch size in ViT. It is important that both the main-block and sub-block sizes are multiples of 8 (or 16 with downsampling) to be equal to the block size of JPEG. Therefore, the main-block and sub-block sizes should be defined on the basis of the block size of JPEG. When the block-size condition is not satisfied, we confirmed that the classification accuracy and compression performance degraded significantly. In other words, the condition allows us to keep the classification accuracy and compression performance high.

JPEG compression generally eliminates image data in the high-frequency component. Therefore, this study suggests that noise added to the high-frequency component has little effect on ViT classification. Additionally, noise-added encrypted images generally have high robustness against attacks. Thus, JPEG noise is also expected to enhance the robustness of EtC images against attacks.

## 5. Conclusions

We investigated the effects of JPEG compression for EtC images on classification results using ViT. JPEG compression never caused severe degradation to the classification accuracy for EtC images; the maximum degradation was 1.22% even when the quality factor was 80. Additionally, the data amount of EtC images was reduced more than 90% under the quality factor. These results proved that JPEG compression for EtC images not only drastically reduced the amount of data but also caused little degradation to the classification accuracy. Further, JPEG compression for plain training images was marginally effective in improving the classification accuracy. Compared with linear quantization, JPEG compression was more effective in terms of the classification and compression performance.

This paper suggests that noise added to the high-frequency component not only keeps the classification accuracy high but also enhances the robustness against attacks. However, the relationships between different types of noise and the classification accuracy or robustness has not been studied in detail. In future work, we will investigate this relationship for more reliable and robust image classification.

## Figures and Tables

**Table 1:** Average amount of image data.

$S_{\mathrm{sb}}$	Transformation Type	Average Amount of Image Data [bpp]						
		JPEG Compression					Linear	No
		Q=100	Q=95	Q=90	Q=85	Q=80	Quantization	Compression
8	Common	4.19	2.08	1.47	1.20	1.04	3.00	24.00
	Independent	5.50	2.80	2.01	1.64	1.42		
16	Common	2.98	1.57	1.13	0.93	0.82		
	Independent	3.49	1.64	1.18	0.98	0.87		
No encryption		2.92	1.54	1.10	0.91	0.80		

**Table 2:** Classification accuracy and change rate for scheme A.

$S_{\mathrm{sb}}$	Transformation Type	Classification Accuracy [%] (Change Rate [%])						
		JPEG Compression					Linear	No
		Q=100	Q=95	Q=90	Q=85	Q=80	Quantization	Compression
8	Common	98.83	98.83	98.80	98.75	98.71	33.29 (66.70)	98.89 (0.00)
		(0.20)	(0.30)	(0.46)	(0.61)	(0.60)		
	Independent	98.45	98.33	98.24	98.00	97.67		
		(0.99)	(1.17)	(1.27)	(1.45)	(1.94)		
16	Common	98.87	98.89	98.89	98.85	98.86		
		(0.12)	(0.18)	(0.17)	(0.19)	(0.25)		
	Independent	98.87	98.86	98.89	98.74	98.66		
		(0.10)	(0.17)	(0.46)	(0.57)	(0.66)		
No encryption for		98.89	98.89	98.81	98.89	98.90		98.89
images and model		(0.08)	(0.10)	(0.18)	(0.25)	(0.23)		(-)

**Table 3:** Classification accuracy for scheme B (Q=85).

$S_{\mathrm{sb}}$	Transformation Type	Classification Accuracy [%]	
		JPEG Compression	Linear Quantization
8	Common	98.84	88.20
	Independent	97.94	
16	Common	98.96	
	Independent	98.80	
No encryption for images and model		98.97	

## Data Availability

The data presented in this study are available on request from the corresponding author.

## References

[1] Konečný J., McMahan H.B., Yu F.X., Richtárik P., Suresh A.T., Bacon D. (2016). Federated learning: Strategies for improving communication efficiency. arXiv.

[2] McMahan B., Moore E., Ramage D., Hampson S., Arcas B.A. Communication-Efficient Learning of Deep Networks from Decentralized Data. Proceedings of the 20th International Conference on Artificial Intelligence and Statistics.

[3] Nagamori H., Kiya H. (2023). Combined Use of Federated Learning and Image Encryption for Privacy-Preserving Image Classification with Vision Transformer. arXiv.

[4] Lou Q., Feng B., Fox G.C., Jiang L. Glyph: Fast and Accurately Training Deep Neural Networks on Encrypted Data. Proceedings of the 34th Conference on Neural Information Processing Systems (NeurIPS 2020).

[5] Boemer F., Lao Y., Cammarota R., Wierzynski C. nGraph-HE: A graph compiler for deep learning on homomorphically encrypted data. Proceedings of the 16th ACM International Conference on Computing Frontiers.

[6] Gilad-Bachrach R., Dowlin N., Laine K., Lauter K., Naehrig M., Wernsing J. Cryptonets: Applying neural networks to encrypted data with high throughput and accuracy. Proceedings of the 33rd International Conference on Machine Learning (PMLR).

[7] Kiya H., Aprilpyone M., Kinoshita Y., Imaizumi S., Shiota S. (2022). An Overview of Compressible and Learnable Image Transformation with Secret Key and Its Applications. APSIPA Trans. Signal Inf. Process..

[8] Aprilpyone M., Kiya H. (2022). Privacy-Preserving Image Classification Using an Isotropic Network. IEEE Multimed..

[9] Aprilpyone M., Kiya H. (2021). Block-Wise Image Transformation With Secret Key for Adversarially Robust Defense. IEEE Trans. Inf. Forensics Secur..

[10] Madono K., Tanaka M., Onishi M., Ogawa T. Block-wise Scrambled Image Recognition Using Adaptation Network. Proceedings of the Workshop on Artificial Intelligence of Things (AAAI WS).

[11] Tanaka M. Learnable image encryption. Proceedings of the IEEE International Conference on Consumer Electronics-Taiwan (ICCE-TW).

[12] Sirichotedumrong W., Maekawa T., Kinoshita Y., Kiya H. Privacy-preserving deep neural networks with pixel-based image encryption considering data augmentation in the encrypted domain. Proceedings of the IEEE International Conference on Image Processing (ICIP).

[13] Yi F., Jeong O., Moon I. (2021). Privacy-Preserving Image Classification With Deep Learning and Double Random Phase Encoding. IEEE Access.

[14] Wang W., Vong C.-M., Yang Y., Wong P.-K. (2017). Encrypted Image Classification Based on Multilayer Extreme Learning Machine. Multidimens. Syst. Signal Process..

[15] Huang Y., Song Z., Li K., Arora S. Instahide: Instance-Hiding Schemes for Private Distributed Learning. Proceedings of the 37th International Conference on Machine Learning (ICML).

[16] Kiya H., IIjima R., Aprilpyone M., Kinoshita Y. (2023). Image and Model Transformation with Secret Key for Vision Transformer. IEICE Trans. Inf. Syst..

[17] Hamano G., Imaizumi S., Kiya H. Image Classification Using Vision Transformer for EtC Images. Proceedings of the Asia-Pacific Signal and Information Processing Association Annual Summit and Conference (APSIPA ASC).

[18] Kurihara K., Kikuchi M., Imaizumi S., Shiota S., Kiya H. (2015). An Encryption-then-Compression System for JPEG/Motion JPEG Standard. IEICE Trans. Fundam..

[19] Ito H., Kinoshita Y., Aprilpyone M., Kiya H. (2021). Image to Perturbation: An Image Transformation Network for Generating Visually Protected Images for Privacy-Preserving Deep Neural Networks. IEEE Access.

[20] Sirichotedumrong W., Kiya H. A GAN-Based Image Transformation Scheme for Privacy-Preserving Deep Neural Networks. Proceedings of the 28th European Signal Processing Conference (EUSIPCO).

[21] Dosovitskiy A., Beyer L., Kolesnikov A., Weissenborn D., Zhai X., Unterthiner T., Dehghani M., Minderer M., Heigold G., Gelly S. An image is worth 16 × 16 words: Transformers for image recognition at scale. Proceedings of the International Conference on Learning Representations (ICLR).

[22] Trockman A., Kolter J.Z. Patches are all you need?. Proceedings of the International Conference on Learning Representations (ICLR).

[23] Weinberger M.J., Seroussi G., Sapiro G. (2000). The LOCO-I lossless image compression algorithm: Principles and standardization into JPEG-LS. IEEE Trans. Image Process..

[24] Vaswani A., Shazeer N., Parmar N., Uszkoreit J., Jones L., Gomez A.N., Kaiser L., Polosukhin I. Attention is all you need. Proceedings of the 31st Conference on Neural Information Processing Systems (NIPS).

[25] Kiya H., Nagamori T., Imaizumi S., Shiota S. (2022). Privacy-Preserving Semantic Segmentation Using Vision Transformer. J. Imaging.

